# Cancer-Associated Fibroblast Density, Prognostic Characteristics, and Recurrence in Head and Neck Squamous Cell Carcinoma: A Meta-Analysis

**DOI:** 10.3389/fonc.2020.565306

**Published:** 2020-11-27

**Authors:** Alexander M. Knops, Andrew South, Ulrich Rodeck, Ubaldo Martinez-Outschoorn, Larry A. Harshyne, Jennifer Johnson, Adam J. Luginbuhl, Joseph M. Curry

**Affiliations:** ^1^Sidney Kimmel Medical College, Thomas Jefferson University, Philadelphia, PA, United States; ^2^Department of Dermatology, Thomas Jefferson University, Philadelphia, PA, United States; ^3^Department of Medical Oncology, Thomas Jefferson University, Philadelphia, PA, United States; ^4^Department of Neurological Surgery, Thomas Jefferson University, Philadelphia, PA, United States; ^5^Department of Otolaryngology—Head and Neck Surgery, Thomas Jefferson University, Philadelphia, PA, United States

**Keywords:** cancer-associated fibroblasts, CAF, tumor microenvironment, myofibroblast, alpha-smooth muscle actin, prognosis, head and neck squamous cell carcinoma

## Abstract

**Introduction:**

The progression and clinical course of head and neck squamous cell carcinoma (HNSCC) relies on complex interactions between cancer and stromal cells in the tumor microenvironment (TME). Among the most abundant of these stromal cells are cancer-associated fibroblasts (CAFs). While their contribution to tumor progression is widely acknowledged, and various CAF-targeted treatments are under development, the relationship between CAF density and the clinicopathologic course of HNSCC has not been clearly defined. Here we examine the published evidence investigating the relationship of cancer-associated fibroblasts to local recurrence and indicators of prognostic significance in HNSCC.

**Methods:**

We conducted a meta-analysis of existing publications that compare the relationship between CAF density, local recurrence, and clinically significant pathologic criteria of disease development (T stage, nodal positivity, clinical stage, vascular invasion, perineural invasion, Ki67 expression, and differentiation). Thirteen studies met the selection criteria, providing a total study population of 926 patients. Forest plots and risk ratios were generated to illustrate overall relationships.

**Results:**

Higher CAF density within the tumor microenvironment is associated with advanced T stage, nodal infiltration, clinical stage, vascular invasion, perineural invasion, Ki67 expression, and differentiation (p <0.05). High CAF density is also associated with increased rates of local recurrence (p <0.001).

**Conclusions:**

Across multiple studies, increased CAF density is correlated with histopathological criteria of poor prognosis in HNSCC. These findings highlight that CAFs may play a pivotal role in HNSCC development and progression. Staining for CAFs may represent a valuable addition to current pathologic analysis and help to guide prognosis and treatment. Understanding the mechanisms by which CAFs reciprocally interact with cancer cells will be crucial for optimization of TME-focused treatment of HNSCC.

## Introduction

Over the last thirty years, multiple lines of evidence have illuminated the importance of the tumor microenvironment (TME) to the development of solid malignancies. Tumor cells and the components of the TME bidirectionally communicate to support neoplastic growth and expansion (1–4). Elements of the TME evolve over time and are reprogrammed to cater for the needs of a growing tumor (5, 6). These observations have changed our understanding of cancer progression and behavior. Once thought to be an isolated group of diseased cells, many malignant tumors, including Head and Neck Squamous Cell Carcinomas (HNSCC), are now regarded as a sophisticated and dynamic tissue consisting of multiple cell types (7–11). Cancer-associated fibroblasts (CAFs) are among the most abundant cells within this tissue.

During tumorigenesis, CAFs develop alongside epithelial cancer cells and adopt a distinct, activated, phenotype (12). As fibroblasts become activated, they express alpha-smooth muscle actin (α-SMA), signifying a myofibroblast phenotype and contractile function, similar to fibroblasts participating in wound healing (13, 14). In solid tumors, historically described as “wounds that do not heal”, these fibroblasts remain persistently activated as CAFs (15). CAFs modulate the TME *via* the secretion of autocrine and paracrine cytokines, as well as extracellular matrix components that provide scaffolds to support the growing tumor cell nests. Many of the classic “hallmarks of malignancy”, as well as emerging hallmarks such as immune evasion and metabolic compartmentalization, are influenced by the presence and functional states of CAFs (6, 16–19).

Similar to other solid cancers, the presence of CAFs in the tumor stroma of HNSCC has been associated with poor survival (20–22). It has been suggested that α-SMA staining is a better predictor for disease mortality than traditional TNM staging (23). However, other studies have not found an association with survival (24, 25).

In this meta-analysis, we sought to synthesize the existing literature to better define the relationship of CAF density on indicators of HNSCC aggressiveness and prognosis as defined by tumor stage and specific histologic findings on tumor biopsy (26, 27). By defining the significance of CAF density within a prognostic framework, utilization of CAFs in clinical medicine can be optimized.

## Material and Methods

**Figure 1:**
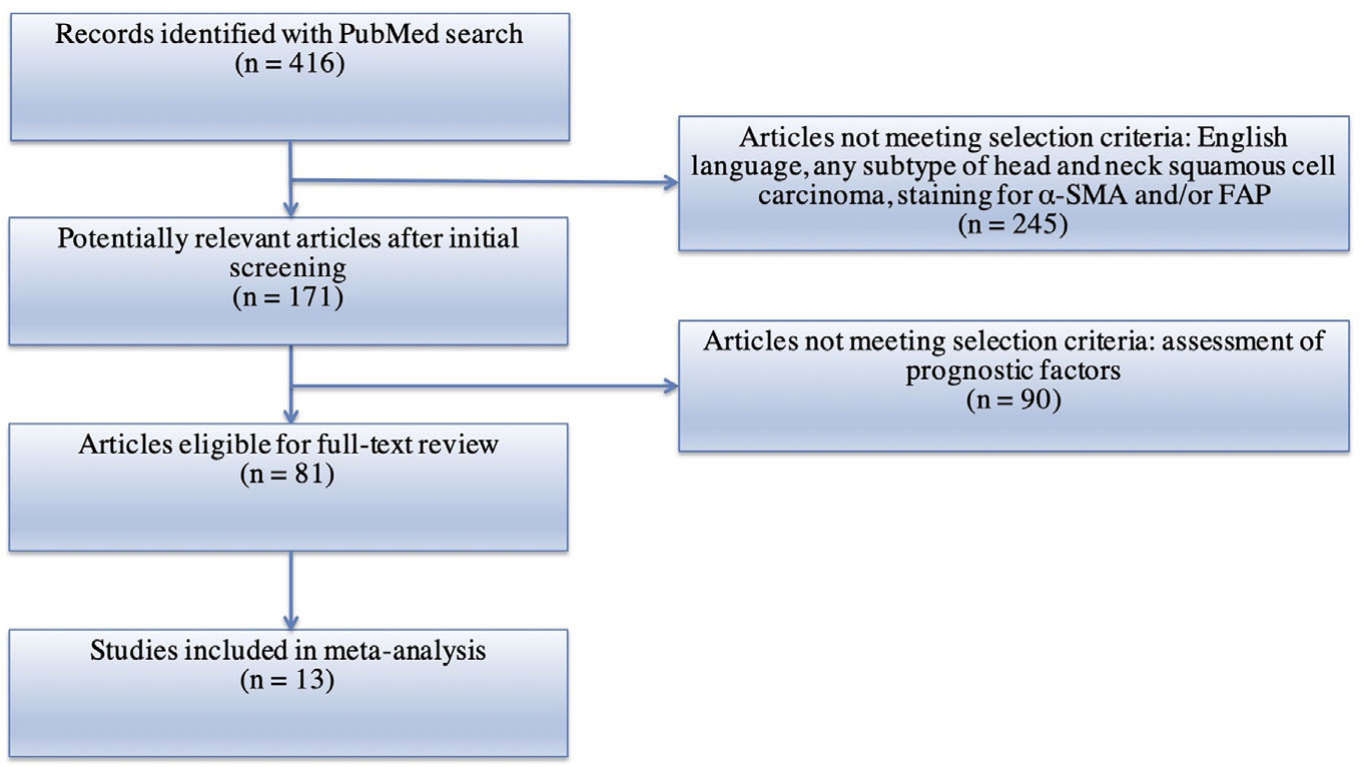
Flowchart of systematic review.

A PubMed search using the following keywords was performed: (“Cancer Associated Fibroblasts” OR “CAFs”) AND (“squamous cell carcinoma”). Preferred Reporting Items Systematic Reviews and Meta-Analyses (PRIMSA) recommendations were followed (28, 29). Articles found from the search were selected for further analysis according to the following criteria: (1) English language; (2) human subjects; (3) squamous cell carcinoma of the head and neck; (4) measurement of CAFs using either alpha-smooth muscle actin (α-SMA) or Fibroblast Activation Protein (FAP) *via* immunohistochemistry; (5) available data on one or more of the following clinical and pathologic characteristics: T stage, N stage, clinical stage, vascular invasion, perineural invasion, Ki67, differentiation, and recurrence. After identification, screening, and determination of eligibility, 13 studies were included in the meta-analysis (**Figure 1**) (22, 24, 25, 28–38). Based on the methods of the selected studies, as well as to facilitate analysis with dichotomous variables, the pathologic markers were grouped in the following manner: advanced T stage included T3 or T4 lesions; nodal positivity included all tumors staged N1–3; clinical stages of III and IV were classified as advanced; poor differentiation was applied to only those samples truly graded as poor; vascular invasion was deemed to be positive or not positive; Ki67 staining was graded as high or low based on a cutoff percentage of positive staining (range: 26–32.4%); perineural invasion was noted to be present or not present; recurrence was either present or not present. No standard scoring system exists to assess the density of CAFs with particular samples. However, all studies included in this analysis used semi-quantitative methods, based on intensity and/or extent of positive staining, to assess and classify CAF density. The methods utilized by each study are noted in **Table 1**. In this analysis, samples with intermediate and high density of CAFs, as determined by the individual study, were grouped together into a high CAF group. This group was compared to a low CAF group, with weak or negative staining in the tumor samples. Correction for multiple testing was not performed. Forest Plots were generated using the Review Manager 5.3 Software (40). Plot specifications were dichotomous for data type, fixed effect for analysis method, and risk ratio for effect measure.

**Table 1:** Characteristics of studies evaluating CAF density and clinicopathologic markers in HNSCC.

Author	Year	Site of Cancer	Number of Patients	Age of Patients (years)	CAF marker used	Prior Therapy Received	Determination of CAF burden in samples
Akrish et al. (25)	2017	Oral SCC	65	28 patients <65, 37 patients >65	α-SMA	No neoadjuvant therapy	• Method proposed by Bello et al. • Based on CAF density
Ding et al. (22)	2014	Oral SCC	50	Mean: 53.5 (26–74)	α-SMA	No neoadjuvant therapy	• Based on CAF density
Fujii et al. (24)	2012	Oral SCC	108	Mean: 66.4 (23–93)	α-SMA	No neoadjuvant therapy	• Modified classification system as described by Kellermann • Based on density and concentration of CAFs
Kellermann et al. (28)	2007	Oral SCC	83	42 patients ≤58, 41 patients >58	α-SMA	No neoadjuvant therapy	• Rated as negative, scanty, or abundant as determined by three independent pathologists
Kellermann et al. (30)	2008	Oral SCC	38	Mean: 61.1 (43–89)	α-SMA	No neoadjuvant therapy	• Rated as negative, scanty, or abundant as determined by three independent pathologists
Liang et al. (31)	2018	Advanced Oral SCC	26	12 patients <50, 14 patients ≥50	α-SMA	Before chemotherapy sample used for meta-analysis	• Based on staining intensity multiplied by percentage of positive staining, Median cutoff between high and low
Lin et al. (32)	2017	Oral SCC	86	26 patients ≤50, 60 patients >50	α-SMA	No neoadjuvant therapy	• Based on staining intensity multiplied by percentage of positive cells (each graded 0–3)
Luksic et al. (33)	2015	Oral SCC	152	26 patients ≤50, 60 patients >50	α-SMA	No neoadjuvant therapy	• Based on Proportion of positive-staining area
Ramos-Vega et al. (34)	2020	Head and Neck SCC	29	Mean: 57 (34–81)	α-SMA	No neoadjuvant therapy	• As described by Fujii et al. • Based on density and concentration of CAFs
Sun et al. (35)	2019	Oral SCC	47	17 patients <60, 30 patients ≥60	α-SMA	No neoadjuvant therapy	• Method described in Cheng et al. (39) • Based on CAF density
Takahashi et al. (36)	2016	Oral SCC	73	17 patients <60, 30 patients ≥60	α-SMA	No neoadjuvant therapy	• According to previously described techniques (Kellermann et al., Fujii et al.) • Based on CAF density
Wang et al. (37)	2019	Oral SCC	121	Mean: 60.2 (34–88)	α-SMA	No neoadjuvant therapy	• Method described by Fujii et al. • Based on density and concentration of CAFs
Zhang et al. (38)	2016	Oral SCC	48	Not reported	α-SMA	No neoadjuvant therapy	• Based on extent of positive staining (high ≥25%)

CAFs, Cancer-Associated Fibroblasts; SCC, Squamous Cell Carcinoma; α-SMA, alpha-Smooth Muscle Actin.

## Results

Characteristics of the 13 studies included in this analysis are shown in **Table 1**. These studies represent a population of 926 patients with surgically resected samples. Twelve studies (897 patients) used samples from oral squamous cell carcinoma, with one of the twelve limiting their analysis to advanced (T3 or T4) oral samples (31). The remaining study evaluated 29 patients with various Head and Neck SCC primary sites including sinonasal, oral, and laryngeal (34). Six studies reported clinical stage according to guidelines from the Union for International Cancer Control and American Joint Committee on Cancer, while three studies reported TNM stage without specifying which staging guidelines were utilized. All studies utilized samples from treatment-naïve patients. All thirteen studies assessed α-SMA expression to determine CAF presence.

### Increased CAF Density Is Associated With Advanced T Stage

**Figure 2:**
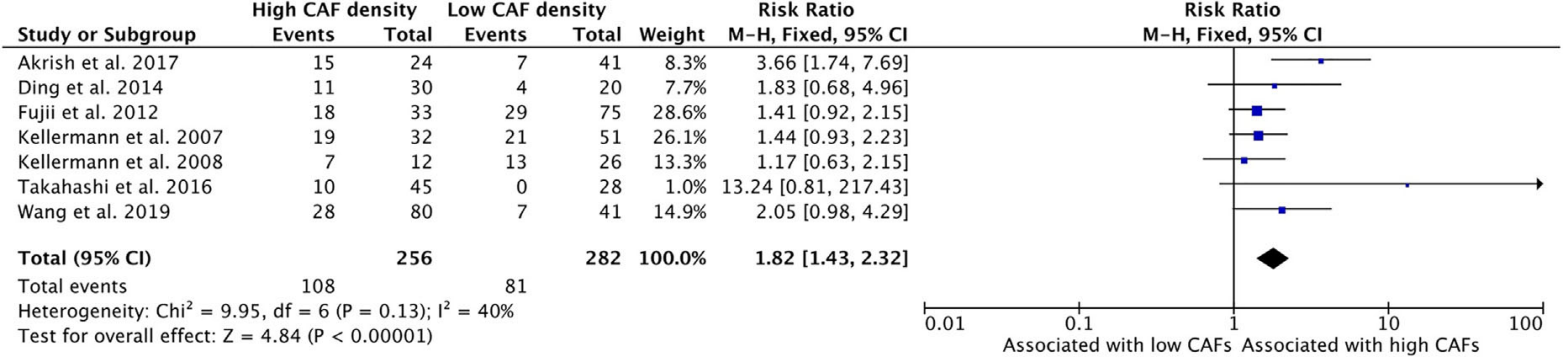
Increased density of CAFs in HNSCC is associated with advanced T stage.

Higher density of CAFs in the tumor microenvironment is associated with stages T3 and T4 HNSCC (**Figure 2**). Seven studies examined the relationship of CAF density and T stage; all seven showed higher density of CAFs was related to increased risk of high T stage tumors. The combined risk ratio of the studies was 1.82 (95%CI = 1.43–2.32). This relationship was statistically significant (p <0.001). The rate of advanced T stage in samples with high CAF density was 42.2%, compared to 28.7% of those with low CAF density.

### Increased CAF Density Is Associated With Lymph Node Metastasis

**Figure 3:**
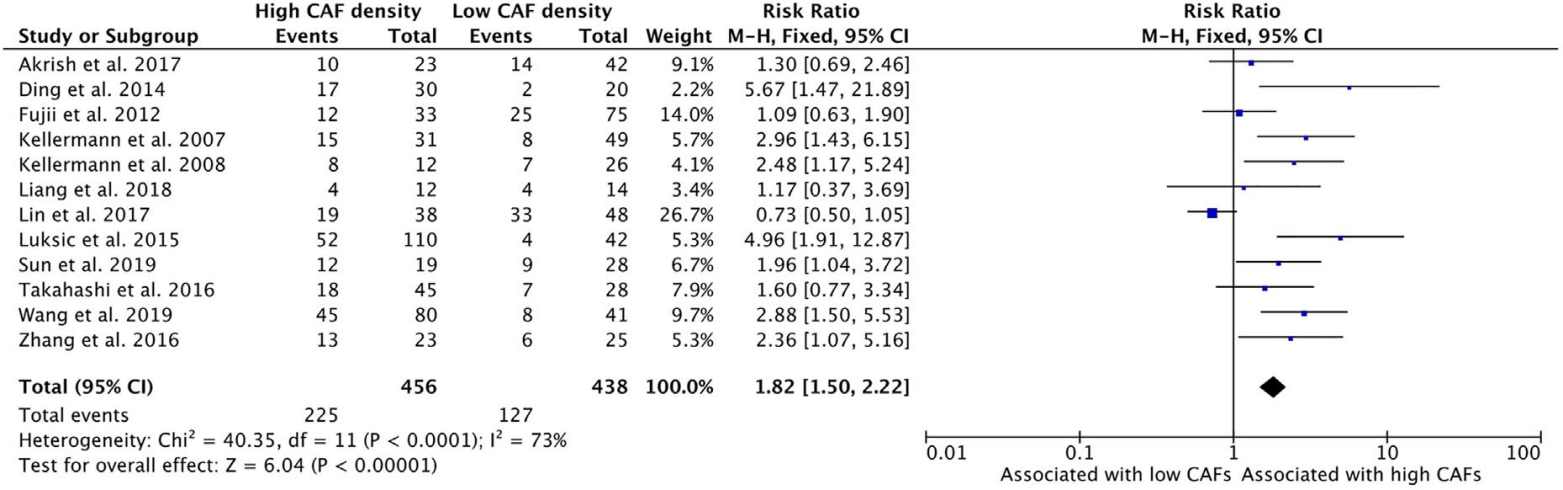
Increased CAF density is associated with lymph node metastasis.

High density of CAFs in the primary tumor TME is associated with increased rates of tumor cell dissemination to regional lymph nodes (**Figure 3**). The relationship of CAFs and nodal infiltration was examined in twelve of the thirteen selected studies and eleven of the studies demonstrated a positive relationship of CAF density and nodal invasion. The cumulative risk ratio of these studies was 1.82 (95%CI = 1.50–2.22, p <0.001). The average rate of nodal positivity in high CAF samples was 49.3%, compared to 29.0% of low CAF tumors.

### Increased CAF Density Is Associated With Advanced Clinical Staging

**Figure 4:**
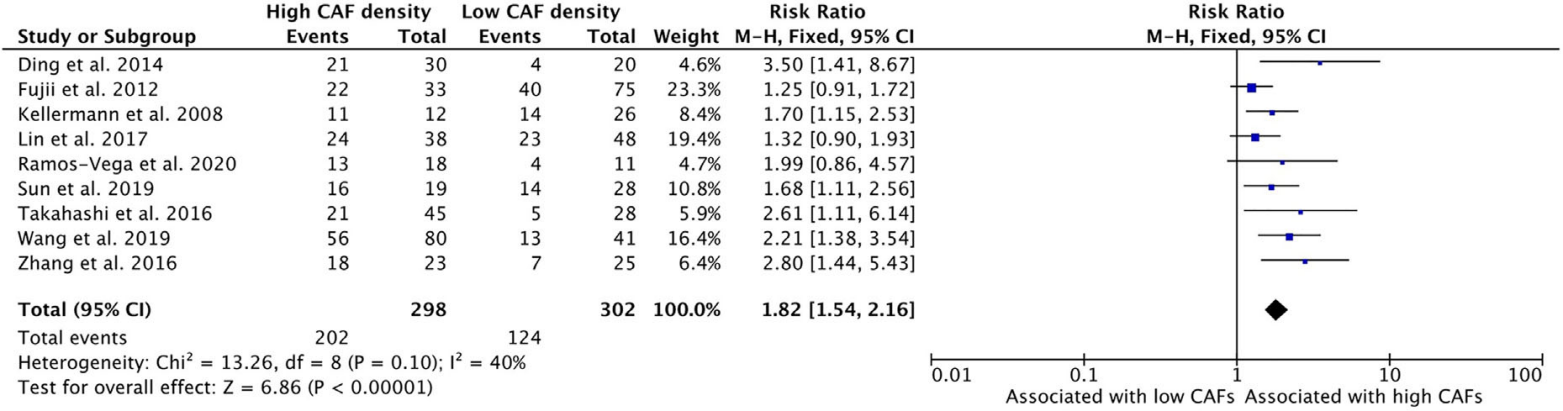
Increased CAF density is associated with high clinical stage.

High density of CAFs within the microenvironment correlated with advanced clinical staging of HNSCC tumors (TNM classification) (**Figure 4**). Nine studies examined this relationship and all nine demonstrated higher TNM classification in those tumors with high CAF density. The cumulative risk ratio was 1.82 (95%CI = 1.54–2.16, p <0.001). The average rate of advanced clinical stage was 67.8% in tumors with high CAF density. In tumors with low CAF density, the average rate was 41.1%.

### Increased CAF Density Is Associated With Higher Rates of Vascular Invasion

**Figure 5:**
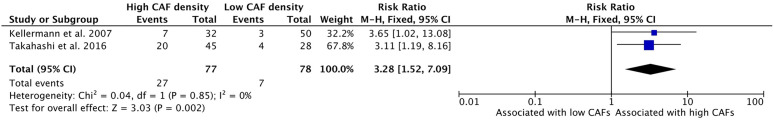
Increased CAF density is associated with high rates of vascular invasion.

Similarly, CAF density is associated with increased rates of vascular invasion (**Figure 5**). Two studies examined this relationship and both showed statistically significant risk ratios. The resulting risk ratio of these studies was 3.28 (95%CI = 1.52–7.09, p = 0.002). In samples that exhibited higher density of CAFs, the average rate of vascular invasion was 35.1% whereas only 9.0% of samples with low CAFs density showed vascular invasion.

### Increased CAF Density Is Associated With Higher Rates of Perineural Invasion

**Figure 6:**
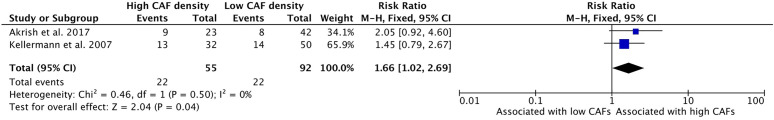
Increased CAF density is associated with high rates of perineural invasion.

Greater density of CAFs in the TME is associated with higher rates of perineural invasion (PNI) in HNSCC (**Figure 6**). Perineural invasion, wherein tumor cells are found to infiltrate the nerve sheath, provides a distinct form of metastatic spread in HNSCC. Two studies examined this relationship, and both showed a positive relationship between CAF density and PNI. 40.0% of samples with high CAF density demonstrated PNI, while 23.9% of samples with low CAF density had PNI upon pathologic examination. These studies had a cumulative risk ratio of 1.66 (95%CI = 1.02–2.69) which was statistically significant (p = 0.04).

### Increased CAF Density Is Associated With Higher Levels of Ki67 Expression

**Figure 7:**
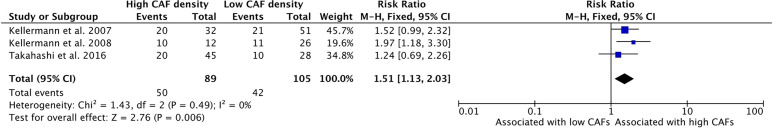
Increased CAF density is associated with increased cellular proliferation, as determined by Ki67 staining.

Higher density of CAFs in the TME is correlated with higher levels of Ki67 expression, a measure of cell proliferation (**Figure 7**). Three studies assessed Ki67 levels within the tumor and all three reported a positive relationship between CAF content and Ki67 staining. Some 56.2% of the total number of samples with high CAF density showed high levels of Ki67, compared to 40.0% of the samples with low CAF density. The combined risk ratio of these studies was 1.51 (95%CI = 1.13–2.03, p = 0.006).

### Increased CAF Density Is Associated With Poor Differentiation

**Figure 8:**
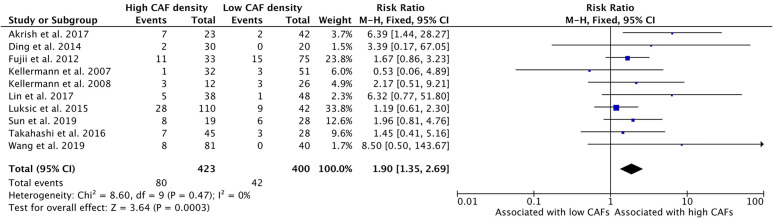
Increased CAF density is associated with poor differentiation of tumor.

High CAF density within the TME is further associated with poor differentiation of HNSCC (**Figure 8**). Ten of the thirteen selected studies determined the relationship between these characteristics; nine of them demonstrated a positive relationship. The average rate of poor differentiation in high CAF density tumors was 19.3%, while low CAF density tumors displayed poor differentiation 13.6% of the time. The resulting risk ratio was 1.90 (95%CI = 1.35–2.69, p <0.001).

### Increased CAF Density Is Associated With Increased Rates of Local Recurrence

**Figure 9:**
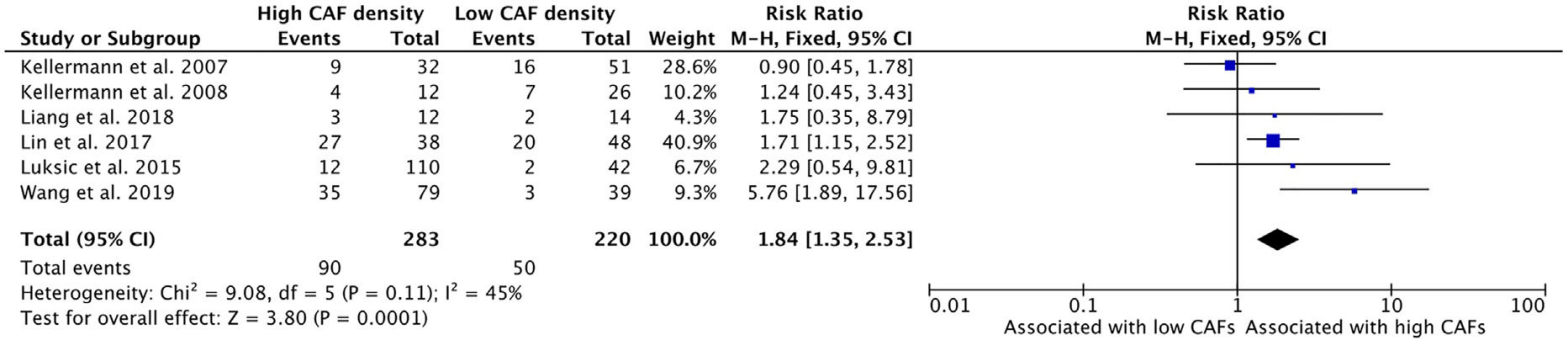
Increased CAF density is associated with local recurrence.

High CAF density within the TME is associated with increased rates of local recurrence of HNSCC (**Figure 9**). Six studies examined this relationship with five studies reporting higher recurrence rates in high CAF density tumors. The cumulative risk ratio was 1.84 (95%CI = 1.35–2.53, P <0.001). The average rate of local recurrence was 31.8% in high CAF density samples, compared to 22.7% in low CAF density samples.

## Discussion

HNSCC, for which mortality remains high despite recent advances in malignancy treatment, demonstrates a remarkable quantity of CAFs, up to 80% of tumor volume in later stage tumors (41, 42). The existing literature has suggested that CAFs negatively affect prognostic characteristics in HNSCC but has not produced a uniform conclusion, with some studies failing to show an association and others claiming a strong positive association (20–25). Two recent meta-analyses have shown that high CAF density is associated with worse overall survival in oral SCC (20, 43). This paper sought to clarify the relationship between CAFs, recurrence, and clinicopathologic markers of significance in HNSCC, including 13 independent studies for a study population of over 900 patients. To our knowledge, this is the first quantitative meta-analysis to examine these relationships. Taken together, the present study shows that, in HNSCC, CAFs are associated with poor prognostic characteristics and increased rates of local recurrence.

This analysis demonstrated that high density of CAFs is associated with higher clinical stage, including T stage and TNM stage (**Figures 2** and **4**). These characteristics were reported by most of the published studies and demonstrated strongly positive risk ratios. Although some publications did not demonstrate a statistically significant relationship, the relationship with T stage and TNM stage was uniform among the studies. This may be attributable to the aforementioned CAF burden in late stage HNSCC, wherein CAFs accumulate and contribute to high proportions of tumor volume.

The presence of high density of CAFs is associated with common modes of tumor spread and metastasis, including perineural invasion, vascular invasion, and lymph node metastasis (**Figures 3**, **5** and **6**). These insidious findings are indicative of a propensity for metastasis and often suggest the need for more aggressive treatment. The relationship between lymph node metastasis and CAF density was not uniform in the literature, with one study showing an inverse, although non-significant, relationship (32). This meta-analysis revealed a strong positive association, with a cumulative risk ratio of 1.82. Vascular invasion and PNI were the least reported finding among the literature, with only two studies remarking on their association with CAFs. Both studies that investigated vascular invasion showed a statistically significant effect of CAFs on vascular involvement. This was reflected in the analysis, with vascular invasion demonstrating the highest risk ratio of all prognostic indicators (3.28), exceeding the 95% confidence interval for all other pathologic variables. Both studies that assessed the relationship with PNI demonstrated increased rates in tumors with high CAF density, however neither demonstrated independent statistical significance between high and low CAF groups. In total, this study suggests that CAFs play a mechanistic role in invasion and correlates with the presence of various forms of cancer spread.

Similarly, high density of CAFs in tumor samples was significantly associated with aggressive histologic features, including poor differentiation and high Ki67 expression (**Figures 7** and **8**). Most studies reported a positive association between CAFs and histologic findings of poor prognosis; however, few studies met statistical significance individually. The relationship between differentiation and CAF density was particularly uncertain in this literature, with only one published study showing a significant relationship (25). However, when summed together, the cumulative literature demonstrated a statistically significant association. The strength of the risk ratios for both poor differentiation and high Ki67 expression were high (1.45 and 1.51, respectively), illustrating a clear association of CAFs with tumor aggressiveness and behavior.

This study showed a significant association of high CAF density with rates of local recurrence (**Figure 9**). Within the literature, this relationship had not been clearly demonstrated, with only two of six studies demonstrating a statistically significant association (32, 37). Our analysis showed a strongly positive risk ratio (1.84), suggesting that CAF density is a clinically meaningful indicator of disease course and outcome.

These findings strongly support the hypothesis that the density of CAFs in HNSCC is associated with multiple poor prognostic factors. Similarly, we demonstrate that high density of CAFs is associated with increased rates of local recurrence. This evidence provides a strong rationale to further investigate the functional relationship of CAFs with carcinoma cells in the tumor microenvironment. Furthermore, this work suggests that staining for CAFs is an indicator of clinical outcomes and may be a valuable addition to existing prognostic strategies.

Immunohistochemical staining for α-SMA was the most commonly utilized method for detecting CAFs in the microenvironment. This allowed for a more homogenous study population and facilitated the comparison between different studies. Other markers for assessing the presence of CAFs have also been suggested in the literature. Higashino et al. performed both FAP and α-SMA staining on esophageal SCC samples and found different staining patterns between the two populations (44). Differences in staining between these markers, and other proposed CAF identifiers, may represent differing origins or functional subtypes of CAFs. The findings in this meta-analysis may be most applicable for α-SMA+ CAF subtypes. Further studies to investigate the differences between these subtypes are needed.

Knowledge of CAFs allows for better understanding of tumorigenesis and increases the feasibility of CAF-directed treatment. Bidirectional interaction between CAFs and tumor cells has been demonstrated in multiple studies and may contribute to the increased tumor aggression and dissemination shown in this meta-analysis.

Although the origin of CAFs in the TME have yet to be clearly defined, the current consensus is that local tissue fibroblasts are activated to become CAFs, under the influence of tumor-derived paracrine factors and cytokines (28, 45, 46). Once activated, CAFs give rise to physical and chemical changes within the microenvironment that facilitate enhanced growth and progression as shown in this data. Through secretion of cytokines like transforming growth factor-beta (TGF-β), CAFs induce a desmoplastic response in the surrounding environment, synthesizing collagen which stiffens the tissue and alters homeostasis (47). The mechanical stress of desmoplasia collapses nearby blood vessels to promote hypoxia, resulting in more aggressive cancer phenotypes. Recurrent malignancy after treatment with radiation exemplifies this phenotype. Orchestrated by CAFs, these recurrences are often exquisitely desmoplastic with extensive collagen cross linking and loss of microvascular structure (48, 49). This modified environment is hypothesized to provide a physical barrier to immune surveillance, enabling unabated tumor growth (50).

Metabolic coupling between CAFs and HNSCC cells further drives tumor development. CAFs demonstrate a “reverse Warburg” effect wherein oxidative stress to tumor cells induces glycolytic behavior and autophagy in nearby CAFs. Catabolites produced in this glycolytic process are then shuttled to tumor cells *via* monocarboxylate transporters (MCTs), allowing for mitochondria-rich tumor cells with high rates of Oxidative Phosphorylation (42, 51). These changes serve to “feed” anabolic tumors cells (52, 53). The findings in this meta-analysis, linking higher CAF density with larger, and more aggressive, primary tumors may reflect this symbiosis.

As noted above, CAFs possess the capacity to directly and indirectly alter the host immune response. CAFs in HNSCC can modulate the function and suppress proliferation of T cells, contributing to the immunosuppressive environment in HNSCC lesions (54, 55). Cytokines secreted by CAFs reportedly promote the attraction of tumor-associated macrophages and skew their differentiation state towards the M2 subtype, which is linked to poor prognosis and outcomes in HNSCC (36, 56, 57). The presence of CAFs may allow tumors to evade immune surveillance and weaken immune response, providing a permissive environment for tumor growth and proliferation.

This meta-analysis demonstrated a strong association between CAF density and tumor invasion. The impact of CAFs on invasion is multifaceted, involving changes to both the TME and cancer cells themselves. It is well documented that CAFs serve to remodel the extracellular matrix and promote “tracks” for invading tumor cells (58). Tumor migration and invasion is supported by CAFs through the secretion of matrix-dissolving proteases including matrix metalloproteins (MMPs) and growth factors including TGF-β (59, 60). Local invasion facilitates dissemination to lymph nodes and neurons, as demonstrated by the increased rates of lymph node positivity and PNI shown in this meta-analysis. Secretion of TGF-β by CAFs may additionally support migration and invasion of tumor cells through robust effects on the epithelial-to-mesenchymal transition (EMT) of HNSCC and enhanced metastatic potential (61).

Prognostication and clinical stratification remain a difficult task in HNSCC. Recent staging modifications by the AJCC have expanded to incorporate extra-nodal extension and depth of invasion into their algorithm (62). However, patients with identical staging may have disparate outcomes. Histopathology may provide valuable data to be incorporated into clinical decision making. Marsh et al. found α-SMA to be the most significant prognostic indicator of all clinical, pathologic, and molecular features (23). Our findings, and recent studies correlating high CAF density with overall survival, support further consideration of CAF utilization within existing prognostic algorithms (20, 43).

The prognostic qualities of CAFs may not be applicable to precancerous lesions. Vered et al. showed a “burst” of CAFs that coincided with the appearance of tongue carcinoma, but not in hyperplastic or dysplastic lesions (63). A study by Etemad-Moghadam et al. demonstrated no α-SMA staining in either normal oral epithelium or oral dysplasia, but all samples of oral carcinoma contained CAFs, to varying degrees (64). This differs from the paradigm seen in other cancer types including colon, breast, and cervical (65–67), wherein α-SMA positive myofibroblasts are present in non-invasive lesions and are thought to promote the progression to carcinoma. Similarly, HPV-positive HNSCC has a unique pathogenesis with different CAF stimulation and activation patterns than those found in HPV-negative disease (68, 69). Further investigation into HPV-associated CAF quantities and function will help to clarify this interaction and guide future treatment.

Tumors with invasive phenotypes or poor differentiation often have a high stromal burden, consisting of large CAF populations. It is important to determine whether differences in prognosis associated with CAF density could be attributable to intrinsic tumor characteristics. To this end, Sun et al. cultured identical SCC lines with either normal fibroblasts or CAFs, demonstrating increased invasion and migration in cell lines co-cultured with CAFs compared to normal fibroblasts (35). Similar findings have been seen in breast cancer experiments (70). These *in vitro* studies offer some insight into the deterministic impact of CAF; however, continued research is necessary to understand how specific CAF and tumor cell interactions result in the prognostic effects shown here.

As the specific impact of CAFs in HNSCC has been better elucidated, CAF-targeted treatment have emerged. For example, inhibitors of Fibroblast Activation Protein (FAP), have shown promising preclinical results in reducing the progression of tumors and promoting anti-tumor immune response (71–73). Similarly, CAF-derived cytokines, like HGF and TGF-β, have been targeted to inhibit tumor aggressiveness and slow resistance to traditional chemotherapy (74, 75). A better understanding of the functional relationship of CAFs and malignant cells is likely to guide identification of additional therapeutic targets in HNSCC.

Further complicating our understanding, CAFs show remarkable heterogeneity and plasticity. Despite underlying commonalities, CAFs within a single tumor may arise *via* different pathways, have different phenotypes, and exhibit functionally different effects on the tumor cells and environment (75–77). This is exemplified by the different expression patterns seen in different methods of CAF identification (44). As recent research has shown, CAF function can be altered by environmental factors (19). Many believe that CAFs, like tumor-associated macrophages, may have either pro-tumoral or anti-tumoral effects depending on subtype or activation (78). This may in part explain why complete α-SMA depletion in mouse models resulted in aggressive pancreatic tumors and reduced survival (79).

Single cell technologies, such as RNA sequencing (scRNA-seq), have recently provided the ability to assess the transcriptional and phenotypical heterogeneity of CAFs. These approaches offer the ability to unmask specific cellular subtypes *via* their transcriptome, providing greater resolution than α-SMA staining. ScRNA-seq research in breast and colon cancer have shown clear delineations between CAF sub-populations (80, 81). Projects that seek to define these subtypes in HNSCC and their prognostic and mechanistic effects are necessary, both for understanding of tumorigenesis and optimization of therapy.

### Limitations

While this meta-analysis demonstrated strong associations between CAF density and indicators of poor prognosis of HNSCC, it was limited in the following ways. First, literature that does not demonstrate significant relationships are far less likely to be published. Because meta-analyses rely on published literature, these findings could be missing unpublished findings that did not meet statistical significance. Additionally, the initial literature search contained multiple studies that examined this relationship but did not present data in a manner amenable to compilation. Thus, we did not include these studies in the statistical analysis. This study demonstrates a correlation between CAF density and prognostic indicators; to determine causality, further investigation would need to be undertaken.

Finally, and as discussed above, the heterogeneity of CAF populations, even within a single tumor, should not be underestimated. Distinct CAF subtypes have been suggested in HNSCC and each subpopulation may hold distinct prognostic significance (76). While this study utilized a broad and encompassing categorization of CAFs, certain subpopulations may have stronger clinical implications.

### Conclusion

**Figure 10:**
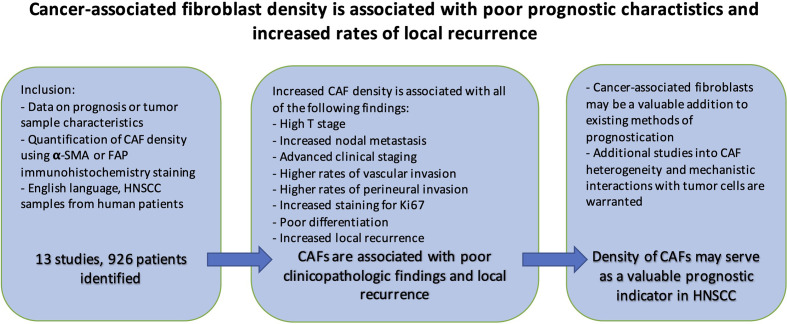
Summary.

CAFs play pivotal roles within the microenvironment of various solid malignancies including HNSCC by aiding tumor invasion, immune suppression, and satisfying the metabolic requirements of rapidly growing tumors (82, 83). This meta-analysis reveals that in HNSCC, the presence of CAFs is associated with poor clinicopathologic features. Samples with high CAF density also demonstrate increased rates of local recurrence (**Figure 10**). Understanding the precise mechanisms, and functional relationship of CAFs with tumor cells will be crucial for optimizing HNSCC treatment. To this end, the functional and structural differences between CAF subtypes and normal fibroblasts need to be more clearly elucidated. The findings support the growing interest in CAFs and their potential to be utilized for clinical stratification and decision-making.

## Data Availability Statement

The raw data supporting the conclusions of this article will be made available by the authors, without undue reservation.

## Author Contributions

JC designed the project. AK performed the literature review, selected papers, collected the data, utilized RevMan to analyze the findings and generate forest plots, and wrote the manuscript. All authors contributed to the article and approved the submitted version.

## Conflict of Interest

The authors declare that the research was conducted in the absence of any commercial or financial relationships that could be construed as a potential conflict of interest.
